# False rifampicin resistant results using Xpert MTB/RIF on urine samples in hospitalised HIV-infected patients

**DOI:** 10.4102/sajhivmed.v20i1.978

**Published:** 2019-08-28

**Authors:** Charlotte Schutz, Amy Ward, Rosie Burton, Mark P. Nicol, Liz Blumenthal, Graeme Meintjes, Andrew D. Kerkhoff

**Affiliations:** 1 Wellcome Centre for Infectious Diseases Research in Africa (CIDRI-Africa), Institute of Infectious Disease and Molecular Medicine (IDM) and Department of Medicine, University of Cape Town, Cape Town, South Africa; 2 GF Jooste Hospital; Department of Medicine, University of Cape Town, Cape Town, South Africa; 3 Division of Medical Microbiology, University of Cape Town and National Health Laboratory Services, Cape Town, South Africa; 4 Division of HIV, Infectious Diseases and Global Medicine at Zuckerberg San Francisco General Hospital and Trauma Center, Department of Medicine, University of California, San Francisco, United States

**Keywords:** HIV, AIDS, Tuberculosis, Xpert, Rifampicin resistance, False resistance

## Abstract

**Background:**

A small proportion of false rifampicin resistant results have previously been reported using GeneXpert MTB/RIF version G4 on sputum samples; however, this has not been investigated for urine samples in HIV-associated tuberculosis (TB).

**Objectives:**

We sought to determine the proportion of false rifampicin resistant results using Xpert MTB/RIF version G4 on urine samples among HIV-infected inpatients investigated for TB.

**Methods:**

Hospitalised HIV-infected patients undergoing systematic TB testing from two cohorts in Cape Town, South Africa, were enrolled. All patients with ≥1 urine Xpert result available were included. Rifampicin resistant urine Xpert results were classified into three mutually exclusive groups: (1) true rifampicin resistance, (2) false rifampicin resistance or (3) unknown after review of available microbiologic and clinical data.

**Results:**

Overall, 1171 patients were included, from whom a total of 1704 urine Xpert results were available on unconcentrated and/or concentrated urine samples. There were 416 samples positive for TB (24.4% [95% CI 22.4–26.5]), of which 43/413 (10.4% [95% CI 7.6–13.8]) were rifampicin resistant (after excluding three results that were falsely positive due to contamination). Of 43 rifampicin resistant Xpert results (among 40 patients), 30 were classified as true resistance, 11 as false resistance and 2 could not be classified. Excluding unclassifiable results, 30/41 results were confirmed as true-positive urine Xpert rifampicin resistance (positive predictive value: 73.2% [95% CI 57.1–85.8]).

**Conclusion:**

Urine Xpert testing showed a high proportion of false rifampicin resistance results. Urine Xpert rifampicin resistant results should be interpreted cautiously and confirmed when possible.

## Introduction

Tuberculosis (TB) remains the leading cause of death in people living with HIV, contributing to one-in-three AIDS-related deaths.^1^ Timely diagnosis of TB in such patients remains challenging because of non-specific presentations and disseminated disease.^2,3,4^ Gene Xpert MTB/RIF, an automated nucleic acid amplification test, is capable of providing results in a few hours and represents an important breakthrough for diagnosing HIV-associated TB. Importantly, Xpert also rapidly detects rifampicin resistance, without need for an additional sample or cartridges. It has been endorsed by the World Health Organization (WHO) since 2010. Sputum Xpert (or Xpert Ultra where available) is currently recommended by the WHO as the initial diagnostic test in patients with suspected HIV-associated TB or multi-drug resistant (MDR) TB.^5^ In those with microbiologically confirmed TB, Xpert MTB/RIF is also recommended by the WHO as a first-line assay for the rapid detection of rifampicin-resistance. It is therefore an important tool in tackling the growing global health challenge of drug resistant (DR)-TB. However, the WHO does not currently have a recommendation regarding the use of Xpert MTB/RIF in urine owing to an insufficient amount of data on the performance and utility of this assay in urine specimens.^6^

In concordance with WHO guidelines, sputum Xpert was implemented as the initial diagnostic evaluation in those with suspected TB and DR-TB in South Africa as well as other countries^7^ and in South Africa, it has now been replaced with the updated Xpert Ultra cartridge. Although Xpert has not been associated with a mortality reduction in most trials to date,^8,9,10^ its implementation has been associated with overall shorter times to starting anti-TB therapy, including DR TB.^8,11,12,13^ It has also increased the diagnostic yield by 1.4% – 15% (compared to sputum microscopy) in clinical trials in Sub-Saharan Africa, Brazil and Indonesia. ^8,9,10,11,13,14,15,16^

Against the backdrop of improved case detection, previous studies have reported on false rifampicin resistance results associated with the Xpert MTB/RIF assay, and meta-analyses found the overall specificity of the Xpert for rifampicin-resistance in sputum samples to be 98% (i.e. 2% showed false rifampicin resistant results) and 99% in extra-pulmonary samples.^17,18^ This however appeared to be associated in part with earlier Xpert cartridge generations.^19^ An implementation study from South Africa found the Xpert G4 cartridge to have excellent positive predictive value for rifampicin resistance of 99.5% (95% CI 98.5–100) in sputum samples.^20^

We have previously found that among HIV-patients requiring acute medical hospitalisation, testing of a single concentrated urine sample detected 2.2 times more TB cases than sputum Xpert testing, largely because of the inability of sick inpatients to produce a sputum sample.^21^ Additionally, a recent randomised, multi-country trial found that the addition of rapid urine-based assays (including urine Xpert) to sputum Xpert testing was associated with reduced mortality among hospitalised HIV-infected patients in sub-group analyses.^22^ This suggests that urine-based testing using Xpert may have an important role in the TB diagnostic algorithm among hospitalised patients with advanced HIV, especially those too ill to produce a sputum sample. However, the proportion of false-positive rifampicin resistance results using Xpert on urine samples has not been reported. We sought to determine the proportion of urine Xpert false rifampicin resistance results among hospitalised HIV-infected patients being investigated for HIV-associated TB in Cape Town, South Africa.

## Methods

### Patients and setting

Patients from two parent cohort studies were included. In the first, patients were recruited at GF Jooste Hospital, South Africa from June 2012 to October 2013. Unselected HIV-infected patients admitted to the medical wards were recruited within 24 h of admission, regardless of TB treatment at the time of admission.^21,23^ GF Jooste Hospital was closed at the end of 2013 and two new hospitals (including Khayelitsha Hospital) were opened serving the same communities at the time the second study was conducted. The second study was undertaken at Khayelitsha Hospital from January 2014 until October 2016 and recruited HIV-infected patients with a low CD4 T-cell count (< 350 cells/µL) admitted to hospital with a suspected new diagnosis of TB. Patients already on TB treatment were excluded from this study. Sputum, blood and urine samples were systematically obtained (when possible) as part of both study protocols and submitted for mycobacteriology (TB culture and/or Xpert). Information about any additional specimens, that were clinically indicated and collected by the medical teams were also recorded – for example, lymph node aspirates, cerebrospinal fluid TB cultures, pleural TB cultures and urine TB cultures.

In the first study, two cases of false urine rifampicin resistance occurred 3 months after study initiation (Appendix Table 1-A1 – patients JTBS097 & JTBS099). Both patients’ urine Xpert samples were collected after a sample was taken from an MDR patient earlier on the same day. It was determined that both samples were likely contaminated due to inadequate cleaning of the reusable bedpan, although laboratory cross-contamination could not be ruled out. We subsequently introduced single-use disposable bed pans (Litha Healthcare Group, Johannesburg, South Africa) and these were used for the remainder of the Jooste Hospital TB study and the duration of the Khayelitsha Hospital TB study. There were no repeat episodes of suspected cross-contamination. Urine was transferred to a polypropylene tube using a sterile syringe. Patients from both cohort studies had urine Xpert testing performed. Demographic details and clinical symptoms were recorded for all patients at study entry. Patients were managed by the hospital and clinic staff, and all TB diagnostic test results were made available by study staff and could be utilised to inform patient care.

### Laboratory methods

Urine Xpert testing for both studies was performed at the Groote Schuur Hospital National Health Laboratory Service laboratory using Xpert MTB/RIF Assay G4 version 5. All specimens were processed using standardised protocols and quality assurance procedures as previously described.^24^ In brief, for the GF Jooste Hospital study, Xpert testing of urine samples was conducted in two ways on each sample. The first method (unconcentrated) utilised 2.0 mL of fresh urine that was centrifuged, resuspended in 0.75 mL phosphate buffer and then tested using Xpert.^25^ The second method (concentrated) used a 30 mL – 40 mL urine sample that was centrifuged at 3000 g for 15 min. The resultant supernatant was removed and the pellet was resuspended in the residual urine volume (without the addition a phosphate buffer); 0.75 mL was then tested using Xpert.^21^ For both methods, Xpert sample reagent (1.5 mL) was added to the samples as per manufacturer’s instructions. The Khayelitsha Hospital study only used Xpert testing on concentrated urine samples and was undertaken using the same methods as described above. The reference standard for drug resistance, including rifampicin resistance for both studies, was a molecular line probe assay (MTBDR plus; Hain Lifescience Nehren, Germany) undertaken on culture isolates from any clinical specimen (not necessarily urine).

### Analysis

Patient populations were from overlapping referral areas in the Cape Town townships and both cohorts included HIV-infected patients requiring medical admission and had detailed TB investigations performed. Urine Xpert rifampicin resistance results were classified by two authors independently by first assessing all available microbiological results (including culture, Xpert and line probe assay) on all clinical samples. In cases where it was not possible to classify urine Xpert rifampicin resistance results by assessing microbiological results from other clinical samples, the type of TB treatment, response to treatment and vital status at 12 weeks were also considered. All patients with urine Xpert rifampicin resistant results were assigned to one of the three mutually exclusive groups: (1) true rifampicin resistant urine Xpert (patients who had rifampicin-resistant TB confirmed by culture or Xpert on other clinical samples) (2) false rifampicin resistant urine Xpert (patients who did not have rifampicin-resistant TB present on additional clinical samples and had a clinical course that was not compatible with drug-resistant TB), (3) unknown (insufficient microbiological and clinical evidence to classify a patient’s urine Xpert rifampicin resistant result). Furthermore, patients with true urine Xpert rifampicin resistance were classified as having heteroresistance if additional independent sample/s from the same clinical episode demonstrated both a rifampicin-susceptible and a rifampicin-resistant *Mycobacterium tuberculosis* (MTB) isolate, i.e. discordant results from two different clinical specimens in the same patient. Two patients (contributing three urine Xpert rifampicin resistance results) were determined to have false urine Xpert rifampicin resistance; this occurred within 3 months of initiating the first study, and was prior to the introduction of single use disposable bedpans (see details above). This led to the introduction of single-use disposable bedpans and avoided further such cases.

### Ethical consideration

Approval for both studies was obtained from the University of Cape Town Faculty of Health Sciences Human Research Ethics Committee and patients provided written informed consent according to the approved study protocols.

## Results

**FIGURE 1:**
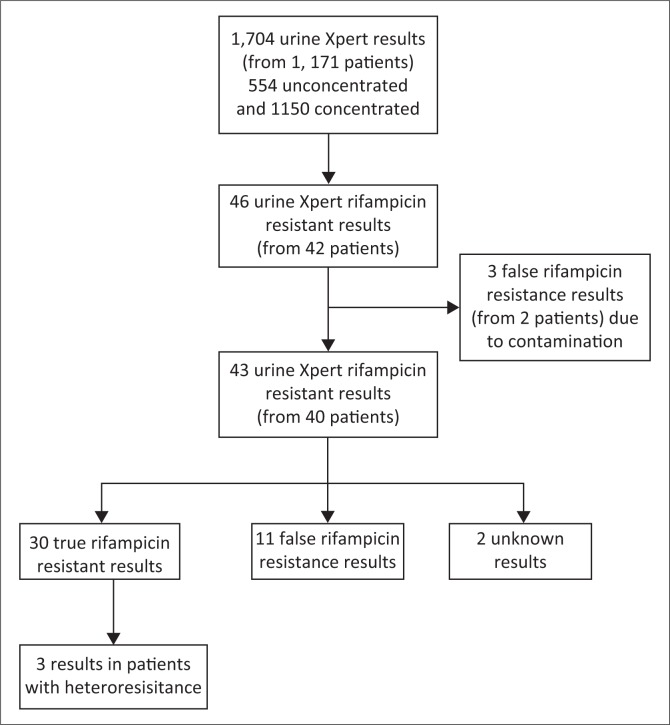
Overview of urine Xpert rifampicin resistance results from two cohorts of hospitalised HIV-patients in Cape Town, South Africa.

There were 585 patients from the GF Jooste Hospital cohort and 586 patients from the Khayelitsha Hospital cohort with urine Xpert results available for a total of 1171 hospitalised HIV-infected patients. Overall 1704 urine Xpert results were available from 1171 patients, of which 554 were performed on unprocessed urine samples and 1150 on concentrated urine samples (Figure 1). Baseline characteristics of the two cohorts were similar. (Table 1).

**TABLE 1:** Baseline characteristics of Jooste Hospital tuberculosis study and Khayelitsha Hospital tuberculosis study patients.

Variable	Jooste Hospital study (*n* = 585)		Khayelitsha Hospital study (*n* = 586)	
	*n*	% or IQR	*n*	% or IQR
**Sex**				
Female	338	57.8	307	52.4
Male	247	42.2	279	47.6
Age, years	35.3	28.9, 41.4	35.9	30.8, 43.9
**ART status**				
Defaulted ART	113	19.3	140	24.1
ART naive	209	35.7	222	38.3
Currently on ART	263	45.0	218	37.6
**TB history**				
Previous TB	263	45.1	268	45.7
Unknown TB history	2	0.3	23	3.9
CD4, cells/mL	134	53, 275	66	24, 138
HIV viral load, log copies/mL	4.2	1.6, 5.5	5.2	3.8, 5.7
Established on TB treatment at enrolment	158	27	-	-

ART, Antiretroviral therapy; TB, Tuberculosis.

Continuous variables presented as median and interquartile range and categorical variables as number and percentage.

Among 1704 urine Xpert results, there were 416 (24.4% [95% CI 22.4–26.5]) samples that tested positive for MTB and 46 results indicating rifampicin resistance among 42 patients (Figure 1). After excluding three results (from two patients) that were determined to be caused by contamination, 43 results from 40 patients remained (*n* = 43/413; prevalence 10.4% [95% CI 7.6–13.8]) and were further classified. The majority of rifampicin resistance results (*n* = 30/43; 69.8% [95% CI 53.9–82.8]) were classified as true urine Xpert rifampicin resistance based on the results from other independent clinical samples. Eleven (11/43, 25.6% [95% CI 13.5–41.2]) results were classified as false rifampicin resistance and two further results (one from each study) could not be classified. Thus, by the most conservative estimate (excluding 2 unknown results), *n* = 30/41 results were confirmed as true urine Xpert rifampicin resistant results, for a positive predictive value of 73.2% (95% CI 57.1–85.8). Comprehensive details for each patient with urine Xpert rifampicin resistance were reported in Appendix Table 1-A1.

**FIGURE 2:**
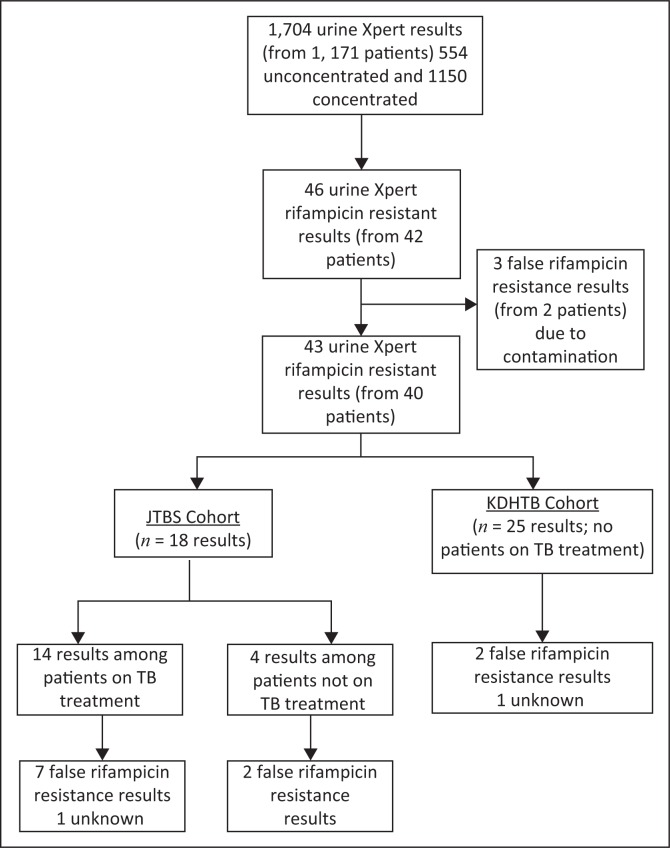
Urine Xpert rifampicin resistance results separated by cohort and tuberculosis treatment status.

False urine rifampicin resistance results were more commonly observed in the Jooste Hospital study: 9/18 (50%) results compared with 2/25 (8%) in the Khayelitsha Hospital study (Figure 2). The Jooste Hospital study enrolled not only patients not yet on TB treatment but also those already established on TB treatment, whereas the Khayelitsha Hospital study excluded patients who were already on TB treatment at the time of admission. In the Jooste Hospital study, there were *n* = 14 results (one unknown rifampicin resistant result) from patients on TB treatment at enrolment and *n* = 4 results from patients not on TB treatment at enrolment and among these, *n* = 7/13 (53.8%) and *n* = 2/4 (50%) had false rifampicin resistant urine Xpert results, respectively. Therefore, in both cohorts and excluding two results that could not be classified, among patients not on TB therapy, *n* = 24/28 (85.7% [95% CI 67.3–96.0] had true positive urine Xpert resistance results compared to *n* = 7/13 (53.8% [95% CI 25.1–80.8] among those receiving TB therapy at study enrolment. This suggests that the positive predictive value of Xpert MTB/RIF for rifampicin resistance is higher among those not on TB treatment compared with those who were already established on TB treatment.

Twelve-week mortality for patients with urine Xpert rifampicin resistant results was 30% (*n* = 12/40) and 7.5% (*n* = 3/40) were lost to follow-up. No deaths were observed among the 10 patients (accounting for 11 results) with false urine Xpert rifampicin resistance. Limited details regarding Xpert probe features for the two patients with false rifampicin resistance in the Khayelitsha Hospital study were available. The clinical microbiologists’ comment for patient KDHTB479 indicated that there was a very low load with a double mutation detected by a delay in probes D and E and that the result was likely false positive. In patient KDHTB439 there was a failure of probe D to bind in the isolate and a repeat sample was requested that demonstrated RIF susceptibility. We were unable to obtain information about the probe features for samples of the JTBS study.

Three patients (*n* = 3/40, 7.5%) with a confirmed rifampicin resistant urine Xpert result had evidence of likely heteroresistant infection. The first patient (Appendix Table 1-A1 – KDHTB203) cultured a drug susceptible isolate from blood (MycoF/lytic bottle), sputum and urine samples but also a rifampicin resistant isolate from sputum during the same admission. The second patient (KDHTB531) cultured a drug-sensitive isolate from blood as well as a drug-resistant isolate from sputum during the same admission. The third patient (JTBS463) was originally started on drug-sensitive TB treatment after a prior sputum Xpert and abscess aspirate culture both showed rifampicin susceptible isolates. One month after starting TB treatment, the patient was admitted for TB immune reconstitution inflammatory syndrome (IRIS). Shortly after discharge, the patient was readmitted for gastroenteritis and was clinically deteriorating despite drug-sensitive TB treatment. At this time, two urine Xpert results showed rifampicin resistance; however, the patient died shortly after receipt of urine Xpert results.

## Discussion

In this study, which included hospitalised HIV-infected patients systematically investigated for TB, the overall proportion of urine Xpert rifampicin resistance results was 10.4% (*n* = 43/413); however, the positive predictive value of urine Xpert MTB/RIF for rifampicin resistance was only 73.2% (*n* = 30/41).

The correct identification of drug-resistant TB has important implications for both the individuals’ health as well as for public health. For the patient, a false rifampicin resistance result may result in not only over-treatment with more toxic drugs that are less efficacious for drug-sensitive TB, but also significantly and unnecessarily prolong treatment times. In high burden, under-resourced settings, a false rifampicin resistance may have important resource implications by resulting in additional drug susceptibility testing, significantly more expensive treatment costs and unnecessary community contact tracing.^26^ Thus, any test that detects DR TB should ideally have very high specificity. Under the best-case scenario when results were restricted to those not receiving TB treatment, we found that Xpert testing of rifampicin resistance on urine samples did not achieve sufficiently high positive predictive value (86%) to be the sole/definitive test for drug-resistant TB identification. This, however, needs to be evaluated in additional settings.

Xpert Ultra is an updated, next-generation sample cartridge for the Xpert platform that is now recommended by the WHO as a replacement for the current Xpert MTB/RIF cartridge^27,28^ and has been implemented in South Africa. It provides increased sensitivity for the detection of MTB in sputum (especially smear-negative and pauci-baciliary disease). Xpert Ultra utilises a new melt curve analysis to detect RIF-resistance; however, its diagnostic accuracy (including specificity) for the detection of rifampicin resistance is similar to that of Xpert.^29^ The results of this study suggest that urine Xpert Ultra rifampicin resistance results should be interpreted cautiously and confirmed by alternative drug susceptibility testing (either phenotypic or alternative genotypic assays) until the specificity of Xpert Ultra for rifampicin resistance detection has been confirmed to be adequately high to warrant stand-alone testing on urine samples.

Of interest, in this cohort we describe three patients with a confirmed urine Xpert rifampicin resistance result who also had drug-sensitive strains from independent samples during the same admission suggesting likely heteroresistance (either polyclonal infection or acquired heteroresistance). The prevalence of heteroresistance in MTB infections has previously been described.^30,31,32^ Although not well-studied, these are likely associated with increased rates of treatment failure for the individual^31^ and could complicate TB control efforts at a population level. Xpert may miss heteroresistance if used as a stand-alone test for the detection of rifampicin resistance, however, early studies show that Xpert Ultra may detect heteroresistance when the resistant DNA comprises 5% or more of the sample.^28^

Strengths of this study include a large number of urine Xpert rifampicin results from two geographically and clinically comparable cohorts where patients were prospectively recruited and underwent systematic testing for TB. Additionally, all TB assays including urine Xpert testing were performed at the same laboratory according to standard protocols. After an error yielded two likely false Xpert rifampicin resistant urine cases due to contamination soon after recruitment initiation, disposable bedpans (single-use) were implemented for the duration of both studies. We therefore recommend that clinicians use single-use specimen collection bedpans and containers when utilising Xpert or Xpert Ultra testing on urine to prevent DNA-cross-contamination between samples.

The reason(s) for the high proportion of false positive urine rifampicin resistance is not entirely clear, but the proportion was higher among those already receiving TB therapy. A limitation of this study is that we did not have data available to systematically evaluate the Xpert probe features associated with our classification of false rifampicin resistance. Different methods of drug susceptibility testing could explain discrepant results in some cases.^33,34^ The majority of drug susceptibility testing on cultured isolates in both studies was PCR-based; however, we also captured results of all TB tests performed in-service and cannot reliably differentiate between drug susceptibility testing performed with other methods such as liquid or solid media for all samples for the duration of the study. Because of the early implementation of disposable bedpans, we do not suspect undetected contamination beyond that described above. Furthermore, because most patients with positive urine rifampicin results did not have paired urine culture isolates available for further genotypic or phenotypic drug-susceptibility testing, patients classified as having false positive rifampicin results may have had heteroresistance with compartmentalised true rifampicin-resistant urinary TB and rifampicin-susceptible TB at other anatomic sites. However, the favourable clinical course of most of these patients on first-line drug-sensitive TB treatment counts against this possibility. Notably, a large proportion of false positive rifampicin results were among those already receiving anti-TB therapy, where 50% of urine Xpert rifampicin resistance results were classified as false resistance; this suggests that further caution should be applied when interpreting urine Xpert rifampicin resistance results in treatment-experienced patients.

An additional limitation of the study is that sequencing of isolates was not performed as part of either study. Sequencing of the rpoB gene would have been particularly useful in the cases that we could not classify as true or false resistance and the heteroresistant cases. Furthermore, urine TB cultures were not routinely performed in either study and it may have been useful to compare drug susceptibility results on isolates cultured from urine samples collected at the same time as the urine Xpert samples.

In conclusion, urine testing using Xpert provides important diagnostic yield for hospitalised HIV-infected patients being investigated for HIV-associated TB, especially in those unable to produce sputum samples. Although the overall proportion of patients with urine Xpert rifampicin resistance in this cohort was relatively low, the proportion of those classified as false rifampicin resistance was substantially higher than has previously been reported on sputum. Urine Xpert rifampicin resistant results should therefore be interpreted with caution, repeated on a second sample in patients at low-risk for drug resistant TB (as currently recommended by the WHO for sputum samples) and confirmed using additional culture-based or molecular assays when possible. Whether these findings apply to Xpert Ultra is an issue that requires further study.

## Appendix 1

**TABLE 1-A1:** Patients with positive urine Xpert rifampicin resistant results – details of additional tuberculosis tests and clinical course.

Study ID	*n*	Sample type	Test	Organism	Susceptibility to rifampicin	Susceptibility to isoniazid	12-week outcome	Classification of urine Xpert rifampicin resistance†	Heteroresistance	Reason for classification
KDHTB067	1	Blood	Culture	MTB	-	-	Died	Unknown	No	No samples could confirm resistance. Patient initially improved on drug-sensitive TB treatment and then experienced rapid neurological deterioration and died on day 3 of therapy.
	1	Blood	Culture	Neg	-	-				
KDHTB086	2	Blood	Culture	Neg	-	-	Died	True Resistance	No	Urine culture sample confirmed rifampicin resistance and sputum Xpert indicated rifampicin resistance.
	1	CSF	Culture	Neg	-	-				
	1	Sputm	Xpert	MTB	Resistant	-				
	1	Urine	Culture	MTB	Resistant	Resistant				
KDHTB110	1	Blood	Culture	Neg	-	-	Alive	True Resistance	No	One blood and three urine culture samples confirmed rifampicin resistance.
	1	Blood	Culture	MTB	Resistant	Resistant				
	1	CSF	Culture	Neg	-	-				
	1	Sputum	Culture	Neg	-	-				
	3	Urine	Culture	MTB	Resistant	Resistant				
KDHTB203	1	Blood	Culture	MTB	-	-	Alive	True Resistance	Yes	Sputum culture result showed rifampicin resistance and three other cultures (sputum, blood and urine) showed drug-sensitive MTB.
	1	Blood	Culture	MTB	Sensitive	Sensitive				
	1	Sputum	Culture	MTB	Sensitive	-				
	1	Sputum	Culture	MTB	Resistant	Sensitive				
	1	Urine	Culture	MTB	Sensitive	Sensitive				
KDHTB238	1	Blood	Culture	MTB	Resistant	Sensitive	LTFU	True Resistance	No	Five cultures and one additional Xpert confirmed rifampicin resistance.
	4	Sputum	Culture	MTB	Resistant	Sensitive				
	1	Sputum	Xpert	MTB	Resistant	-				
KDHTB242	1	Blood	Culture	Neg	-	-	Alive	True Resistance	No	Four cultures confirmed rifampicin resistance.
	1	Pleural	Culture	MTB	Resistant	Sensitive				
	2	Sputum	Culture	MTB	Resistant	Sensitive				
	1	Urine	Culture	MTB	Resistant	Sensitive				
KDHTB264	2	Blood	Culture	Neg	-	-	Alive	True Resistance	No	One culture result confirmed rifampicin resistance.
	1	CSF	Culture	Neg	-	-				
	1	Pleural	Culture	MTB	Resistant	Resistant				
	2	Urine	Culture	Neg	-	-				
KDHTB398	2	Blood	Culture	MTB	Resistant	Resistant	Died	True Resistance	No	Two culture results confirmed rifampicin resistance.
	1	CSF	Culture	Neg	-	-				
KDHTB416	1	Blood	Culture	Neg	-	-	LTFU	True Resistance	No	Two culture results confirmed rifampicin resistance.
	1	Sputum	Culture	Neg	-	-				
	2	Sputum	Culture	MTB	Resistant	Resistant				
KDHTB439	1	Blood	Culture	Neg	-	-	Alive	False Resistance	No	One culture and three Xpert results showed rifampicin sensitivity.
	1	Pleural	Xpert	MTB	Sensitive	-				
	1	Sputum	Xpert	MTB	Sensitive	-				
	1	Sputum	Culture	MTB	Sensitive	Sensitive				
	1	Urine	Culture	Contam	-	-				
	1	Urine	Xpert	MTB	Sensitive	-				
KDHTB444	2	Blood	Culture	MTB	-	-	Died	True Resistance	No	One culture result confirmed rifampicin resistance.
	1	Blood	Culture	MTB	Neg	-				
	1	Sputum	Culture	MTB	Resistant	Resistant				
KDHTB445	1	Blood	Culture	Neg	-	-	Alive	True Resistance	No	Three culture results confirmed rifampicin resistance.
	3	Sputum	Culture	MTB	Resistant	Resistant				
KDHTB477	1	Blood	Culture	MTB	Resistant	Sensitive	Alive	True Resistance	No	Three culture results confirmed rifampicin resistance.
	2	Sputum	Culture	MTB	Resistant	Sensitive				
KDHTB479	1	Blood	Culture	Neg	-	-	Alive	False Resistance	No	Two culture results showed rifampicin sensitivity.
	2	Sputum	Culture	MTB	Sensitive	Sensitive				
KDHTB524	1	Blood	Culture	Neg	-	-	Alive	True Resistance	No	One culture and two Xpert results confirmed rifampicin resistance.
	1	Sputum	Culture	Neg	-	-				
	2	Sputum	Xpert	MTB	Resistant	-				
	1	Sputum	Culture	MTB	Resistant	Sensitive				
KDHTB531	1	Blood	Culture	MTB	Sensitive	Sensitive	Died	True Resistance	Yes	One culture result confirmed rifampicin resistance and one showed rifampicin sensitivity.
	1	Sputum	Culture	MTB	Resistant	Sensitive				
KDHTB555	1	Blood	Culture	MTB	Resistant	Resistant	Alive	True Resistance	No	Three culture results confirmed rifampicin resistance.
	2	Blood	Culture	Neg	-	-				
	2	Sputum	Culture	MTB	Resistant	Resistant				
KDHTB556	1	Abscess	Culture	MTB	Resistant	Resistant	Died	True Resistance	No	Two culture results confirmed rifampicin resistance.
	1	Blood	Culture	Neg	-	-				
	1	Sputum	Culture	MTB	Resistant	Resistant				
KDHTB560	2	Blood	Culture	Neg	-	-	Alive	True Resistance	No	One culture result confirmed rifampicin resistance.
	1	Sputum	Culture	MTB	Resistant	Resistant				
KDHTB598	1	Blood	Culture	MTB	Resistant	Sensitive	Died	True Resistance	No	Three culture results confirmed rifampicin resistance.
	1	CSF	Culture	MTB	Resistant	Sensitive				
	1	Sputum	Culture	MTB	Resistant	Sensitive				
KDHTB616	1	Abscess	Culture	MTB	Resistant	Sensitive	Alive	True Resistance	No	Four culture results confirmed rifampicin resistance.
	2	Blood	Culture	MTB	Resistant	Sensitive				
	5	Blood	Culture	Neg	-	-				
	1	Blood	Culture	MTB	Resistant	Sensitive				
KDHTB627	1	Blood	Culture	MTB	Resistant	Sensitive	Died	True Resistance	No	One culture result confirmed rifampicin resistance.
KDHTB631	1	Blood	Culture	Neg	-	-	Alive	True Resistance	No	One Xpert result and two culture results confirmed rifampicin resistance.
	2	Sputum	Culture	MTB	Resistant	Resistant				
	1	Sputum	Xpert	MTB	Resistant	-				
	1	Urine	Culture	Neg	-	-				
KDHTB660	6	Blood	Culture	Neg	-	-	Died	True Resistance	No	Two culture results confirmed rifampicin resistance.
	2	Blood	Culture	MTB	Resistant	Sensitive				
KDHTB668	1	Blood	Culture	Neg	-	-	Alive	True Resistance	No	Treated for MDR-TB, improved and survived.
JTBS021	1	Blood	Culture	Neg	-	-	Alive	Unknown	No	No samples confirmed rifampicin resistance. Patient started drug-sensitive TB treatment. Inadequate information on improvement or deterioration.
	1	Pleural eff	Culture	Neg	-	-				
JTBS074	1	Urine	Xpert	Neg	-	-	Alive	False Resistance	No	No sample confirmed resistance and patient improved on drug-sensitive TB treatment.
	1	Blood	Culture	Neg	-	-				
	1	CSF	Culture	Neg	-	-				
JTBS090	2	Urine	Culture	MTB	Resistant	Resistant	LTFU	True Resistance	No	Three culture results confirmed rifampicin resistance.
	1	Sputum	Culture	MTB	Resistant	Resistant				
JTBS097†	2	Blood	Culture	MTB	Sensitive	Sensitive	Died	Contamination	No	Two culture results showed rifampicin sensitivity. Patient started drug-sensitive TB treatment, then switched to MDR-TB treatment on basis of urine Xpert result; however, died before other drug susceptibility results were available.
	1	Sputum	Culture	Neg	-	-				
	1	Sputum	Culture	MTB	Sensitive	Sensitive				
	1	Urine	Culture	Neg	-	-				
	1	Sputum	Culture	Contam	-	-				
	1	CSF	Culture	Contam	-	-				
JTBS098	1	Urine	Xpert	Neg	-	-	Died	True Resistance	No	No samples from enrolment admission confirmed rifampicin resistance; however, a 7-month-old result showed rifampicin resistance on pleural fluid, which was untreated. It is unclear why the patient was not treated for MDR-TB and he died shortly after admission to hospital.
	1	Blood	Culture	Neg	-	-				
	2	CSF	Culture	Neg	-	-				
	1	Sputum	Culture	Contam	-	-				
JTBS099†	4	Tracheal aspirate	Culture	MTB	Sensitive	Resistant	Alive	Contamination	No	Four culture results showed rifampicin sensitivity and patient responded to drug-sensitive TB treatment.
JTBS159	1	Urine	Xpert	Neg	-	-	Alive	False Resistance	No	No cultures confirmed rifampicin resistance and one sputum Xpert showed rifampicin sensitivity. Patient treated with drug-sensitive TB treatment and improved.
	1	Blood	Culture	Neg	-	-				
	2	Sputum	Culture	Neg	-	-				
	1	Sputum	Xpert	MTB	Sensitive	-				
	1	Sputum	Culture	Contam	-	-				
	1	Urine	Culture	Neg	-	-				
JTBS160	1	Blood	Culture	Neg	-	-	Alive	False Resistance (on two results)	No	Two cultures showed rifampicin sensitivity and patient improved on drug-sensitive TB treatment.
	2	Sputum	Culture	MTB	Sensitive	Sensitive				
	1	Urine	Culture	Neg	-	-				
JTBS181	1	Urine	Xpert	MTB	Resistant	-	Alive	True Resistance (on two results)	No	Two culture results confirmed rifampicin resistance.
	2	Blood	Culture	Neg	-	-				
	1	Sputum	Culture	MTB	Resistant	-				
	2	Ascites	Culture	Neg	-	-				
	1	Urine	Culture	MTB	Resistant	Resistant				
JTBS192	1	Sputum	Culture	MTB	Resistant	-	Died	True Resistance	No	Three culture results confirmed rifampicin resistance.
	1	Urine	Xpert	Neg	-	-				
	1	Urine	Culture	MTB	Resistant	Resistant				
	1	Sputum	Culture	MTB	Resistant	Resistant				
	1	Sputum	Culture	Contam	-	-				
	1	Sputum	Culture	Neg	-	-				
JTBS202	1	Urine	Xpert	Neg	-	-	Alive	False Resistance	No	One culture result showed rifampicin sensitivity and patient improved on drug-sensitive TB treatment
	1	Blood	Culture	Neg	-	-				
	1	Sputum	Culture	Neg	-	-				
	1	Sputum	Culture	MTB	Sensitive	Sensitive				
JTBS211	9	Sputum	Culture	Neg	-	-	Alive	False Resistance	No	No confirmation of rifampicin resistance patient improved on drug-sensitive TB treatment.
	1	Sputum	Culture	Contam	-	-				
JTBS249	1	Urine	Xpert	Neg	-	-	Alive	False Resistance	No	No confirmation of rifampicin resistance and patient improved on drug-sensitive TB treatment.
	1	Blood	Xpert	Neg	-	-				
	5	CSF	Culture	Neg						
JTBS358	1	Urine	Xpert	MTB	Sensitive	-	Alive	False Resistance	No	No cultures confirmed rifampicin resistance and patient improved on drug-sensitive TB treatment.
	1	Urine	Xpert	Neg	-	-				
	1	Sputum	Culture	MTB	-	-				
	1	Blood	Culture	Neg	-	-				
JTBS463	1	Urine	Xpert	MTB	Resistant	-	Died	True Resistance (from two results)	Yes	Two urine Xpert results showed rifampicin resistance, but one abscess culture and one sputum Xpert (2 months prior) showed rifampicin sensitivity. Patient started on drug-sensitive TB treatment. About 1 month after starting TB treatment the patient was admitted for TB IRIS and anaemia. Patient was then readmitted shortly thereafter with gastroenteritis and was deteriorating despite drug-sensitive TB treatment; it was at this time two urine Xpert results showed rifampicin resistance; however the patient died shortly after receipt of urine Xpert results.
	1	Abscess	Culture	MTB	Sensitive	Sensitive				
JTBS534	1	Urine	Xpert	Neg	-	-	Died	True Resistance	No	No samples confirmed rifampicin resistance. Patient presented with ascites and pleural fluid of which all TB tests were negative. Ascitic fluid had high ADA and patient was started on drug-sensitive TB treatment. About 4 weeks later, the patient was readmitted while clinically deteriorating on drug-sensitive TB treatment. A urine Xpert result showed rifampicin resistance; however, the patient died shortly after receipt of urine Xpert result.
	1	Blood	Culture	Neg	-	-				
	1	Ascites	Culture	Neg	-	-				
	1	Pleural	Culture	Neg	-	-				
	1	Sputum	Culture	Neg	-	-				
JTBS574	1	Urine	Xpert	Neg	-	-	Alive	False Resistance	No	One culture and two Xpert results showed rifampicin sensitivity and patient improved on drug-sensitive TB treatment.
	1	Blood	Culture	Neg	-	-				
	1	Sputum	Culture	MTB	Sensitive	Sensitive				
	2	Sputum	Xpert	MTB	Sensitive	-				

*n* = number of samples; – Susceptibility unknown or not available. Neg, negative; Contam, Contaminated; MTB, Mycobacterial tuberculosis; LTFU, lost to follow-up; TB, tuberculosis; Pleural, Pleural effusion; Abscess, Abscess aspirate; Drug-sensitive TB treatment = Intensive phase with isoniazid, rifampicin, ethambutol and pyrazinamide, followed by consolidation phase with isoniazid and rifampicin.

† , Two patients had false urine Xpert rifampicin resistance due to contamination (accounting for three results). These patients were excluded from analysis.

For JTBS patients, nearly all had their urine specimens tested by Xpert using two different methods (unconcentrated and concentrated). This accounts for why patients may have two urine Xpert results. However, in the table above, rifampicin resistance was only detected on one urine Xpert result, unless stated otherwise. Some patients may have had an additional urine Xpert performed for confirmation after receiving a rifampicin resistant result.

Of note, the table includes all other TB cultures and Xperts conducted on all clinical specimens, including repeat urine Xpert tests conducted.
